# Clinical and radiological features of a case of primary encephalitis induced by SARS-CoV-2 omicron variant infection: A case report

**DOI:** 10.1097/MD.0000000000035229

**Published:** 2023-09-15

**Authors:** Xiong Zhang, Ruiting Hu, Fanyu Zhao

**Affiliations:** a Department of Radiology, Minzu Hospital of Guangxi Zhuang Autonomous Region, Nanning, Guangxi, China.

**Keywords:** case report, COVID-19, encephalitis, MRI, omicron

## Abstract

**Rationale:**

In 2022, severe acute respiratory syndrome coronavirus 2 (SARS-CoV-2) Omicron spread widely around the world. In the context of most literature reporting weakened virulence of the virus, immunocompromised patients who have not been vaccinated should be vigilant for the development of encephalitis following SARS-CoV-2 infection.

**Patient concerns:**

A 58-year-old male patient with immunodeficiency presented with respiratory and psychiatric symptoms after contracting SARS-CoV-2 Omicron variant.

**Diagnoses:**

The patient was diagnosed with coronavirus disease 2019 infection and associated acute primary encephalitis.

**Interventions:**

The patient was received comprehensive treatment including Azvudine antiviral therapy, immunoglobulin infusion, and methylprednisolone anti-inflammatory therapy.

**Outcomes:**

The patient’s condition improved and he was discharged smoothly. One month after discharge, the patient returned for follow-up, and the occipital lobe still had a few slow waves on electroencephalogram, but the patient reported no seizure events since discharge.

**Lessons:**

During the prevalence of the SARS-CoV-2 Omicron variant, we believe that it is still necessary to be vigilant about immunocompromised patients developing encephalitis. Early use of cranial magnetic resonance imaging as a diagnostic assistance is conducive to early diagnosis and treatment of patients.

## 1. Introduction

In 2022, the severe acute respiratory syndrome coronavirus 2 (SARS-CoV-2) Omicron variant caused a widespread epidemic around the world. This strain of the virus has a high transmission rate and infectivity,^[1]^ but generally causes milder symptoms. Most patients only exhibit fever and coughing, while a small percentage experience extrapulmonary symptoms such as headache, diarrhea, muscle aches, loss of smell, and loss of taste.^[2]^ Only a very small number of patients experience neurological complications, and cases where the virus directly invades the central nervous system are extremely rare.^[3,4]^ This article reports a case of acute viral encephalitis caused by SARS-CoV-2 Omicron variant infection. The patient presented with headache, seizures, episodic vision loss, and hallucinations, magnetic resonance imaging (MRI) examination showed abnormal signal shadows in the cerebral lobes, and the cerebrospinal fluid (CSF) macro-gene detection showed a positive result for SARS-Cov-2 (with a sequence number of 6). After 1 month of treatment, the patient had no headache, seizures, vision returned to normal, no hallucinations, and he was discharged. This article provides a detailed description of the patient’s clinical symptoms, changes in imaging examination, and treatment process, which can serve as a reference for the diagnosis and treatment of coronavirus disease 2019 (COVID-19)-induced encephalitis in clinical practice.

## 2. Case report

**Figure 1.**
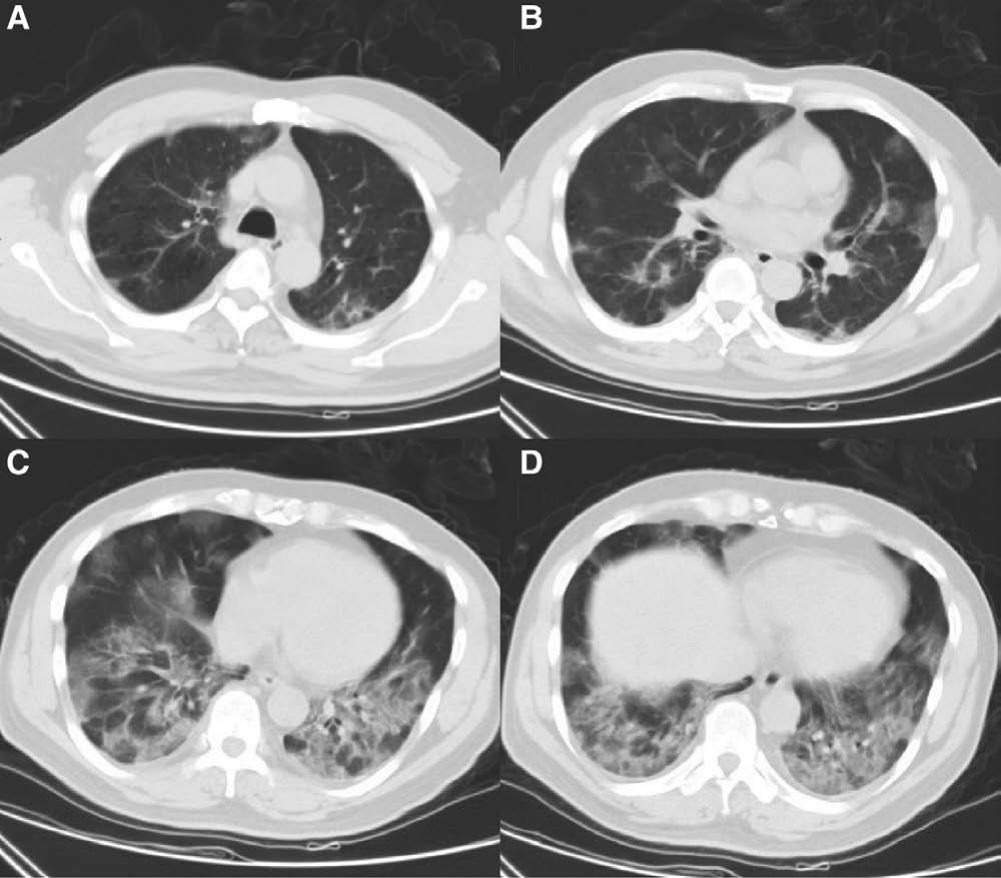
The initial chest CT scan of the patient on the 1th day of admission (A–D): showed multiple “ground glass” opacities in both lungs. CT = computed tomography.

**Figure 2.**
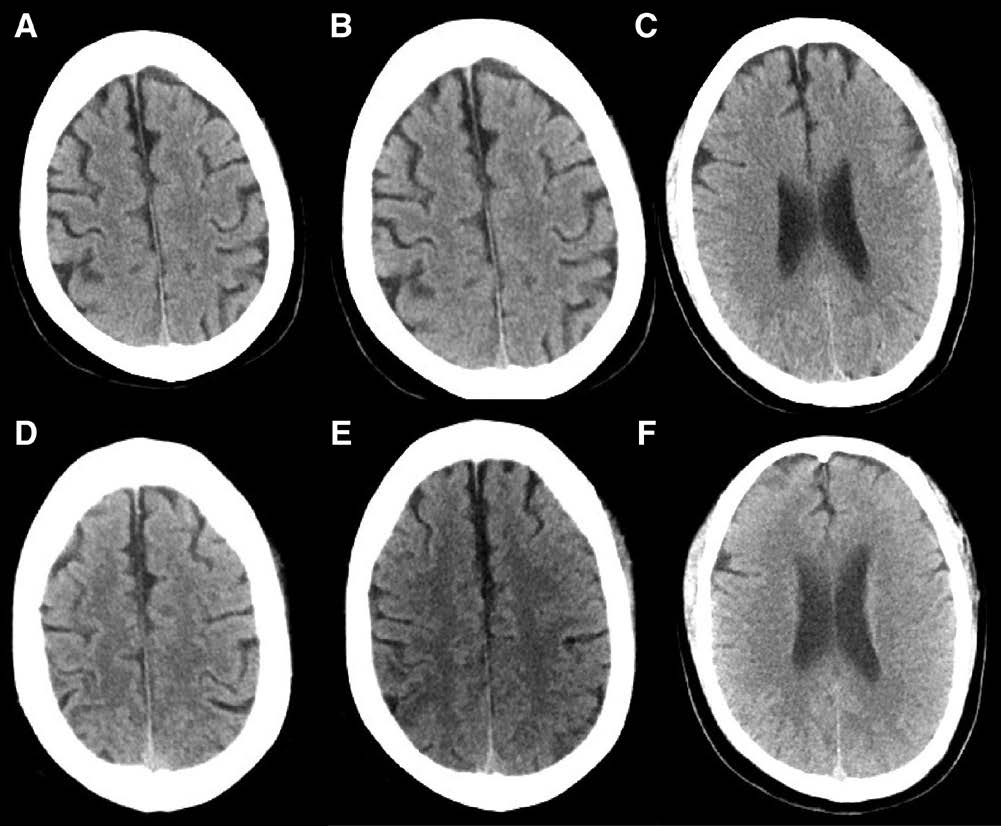
First cranial CT scan of the patient on the 7th day of admission (A–C): no obvious abnormalities were found; second cranial CT scan on the 11th day of admission (D–F): local density reduction in right parietal lobes may be seen indistinctly. CT = computed tomography.

**Figure 3.**
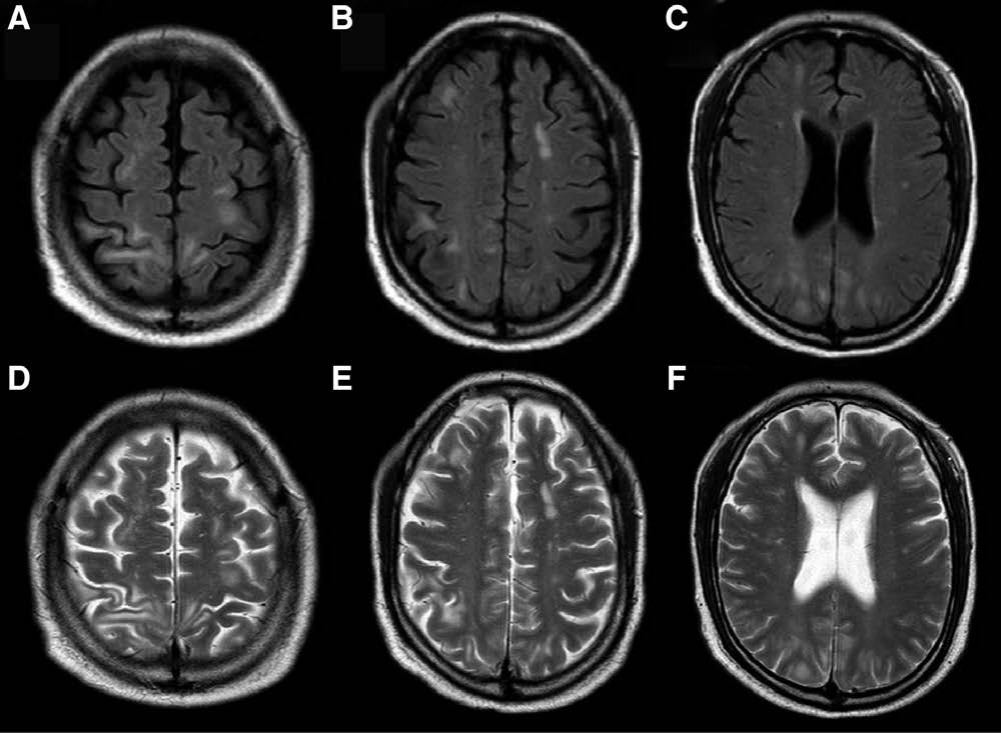
The patient’s first cranial MRI examination on the 14th day of admission (A–C) FLAIR sequence, (D–F) T2WI sequence: multiple patchy and gyriform FLAIR and T2WI hyperintense lesions in the gray matter and gray-white matter junctions of both frontal, parietal, and occipital lobes, as well as high signal intensity in T2WI. FLAIR = fluid attenuated inversion recovery, MRI = magnetic resonance imaging, T2WI = T2 weighted imaging.

**Figure 4.**
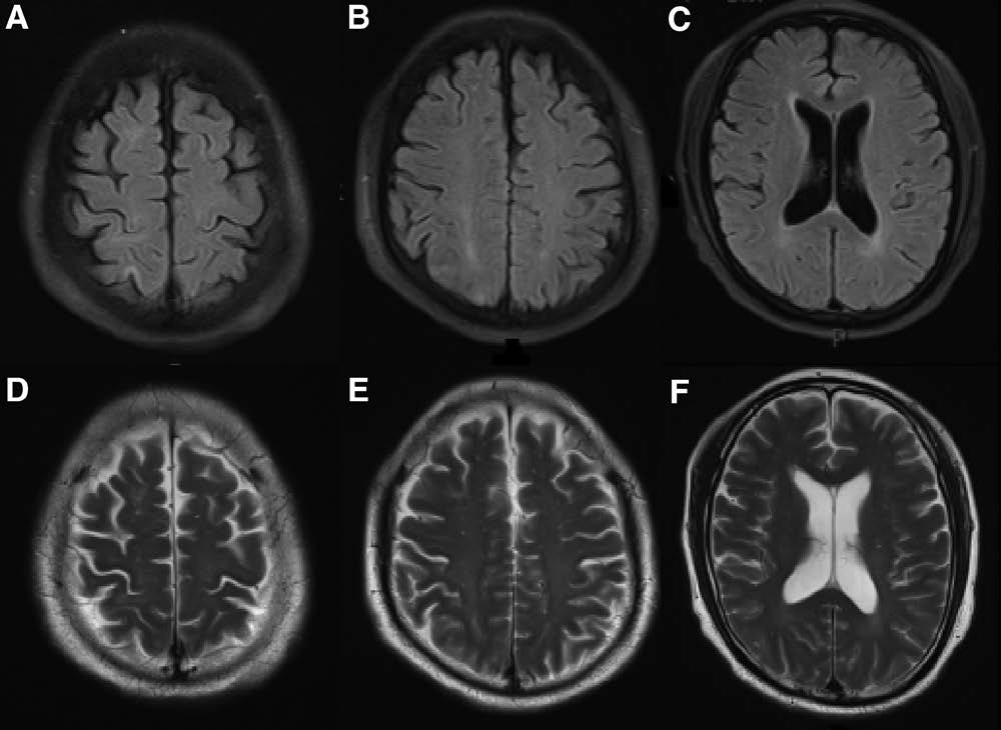
The patient’s second cranial MRI scan on the 25th day of admission (A–C) FLAIR sequence, (D–F) T2WI sequence: only scattered small patchy FLAIR and T2WI high signal changes in the right frontal and parietal lobes. Compared to the first scan, most of the high signal changes in the bilateral frontal, parietal, and occipital lobes had disappeared. The patient’s symptoms improved significantly. FLAIR = fluid attenuated inversion recovery, MRI = magnetic resonance imaging, T2WI = T2 weighted imaging.

In December 2022, a 58-year-old male patient was admitted to the hospital where the author works due to “fever, shortness of breath, and headache for 4 days.” Upon admission, the patient had a temperature of 39.3ºC, accompanied by chills, cough, shortness of breath, and headache. The patient reported no history of COVID-19 vaccination and no previous infection with SARS-CoV-2. The patient had a history of systemic lupus erythematosus. Due to poor compliance and non-standardized diagnosis and treatment, he had received prednisone intermittently, but had been discontinued for more than 1 year. He usually have leukopenia (1.9–2.5 × 10^9^/L) (the reference range is 3.5–9.5 × 10^9^/L). Upon admission, a blood routine examination showed white blood cells was 2.07 × 10^9^/L (the reference range of our hospital is 3.5–9.5 × 10^9^/L), red blood cells was 4.11 × 10^12^/L (the reference range is 4.3–5.8 × 10^9^/L), platelets was 79 × 10^9^/L (the reference range is 125–350 × 10^9^/L), and absolute lymphocyte count was 0.26 × 10^9^/L (the reference range is 1.1–3.2 × 10^9^/L). Tuberculosis antibodies and cryptococcal antigens were negative. antinuclear antibodies were positive at a titer of 1:100 with a speckled pattern. The anti-immunoglobulin G and anti-C3d test results were positive. The levels of immunoglobulin and complement were normal. Immunological function testing revealed a significantly lower than normal total T lymphocyte count of 221.02 cells/μL, an auxiliary T lymphocyte count of 157.19 cells/μL, and a killer T lymphocyte count of 62.24 cells/μL. Cytokine monitoring showed that IL-6 was 2.32 pg/mL, which was within the normal range. Pharyngeal swab nucleic acid testing for COVID-19 showed positive results for the ORF-lab gene and N gene of the novel coronavirus. Chest computed tomography (CT) revealed multifocal ground-glass opacities in both lungs, the lesions were mostly distributed in the periphery of the lungs or under the pleura, suggesting viral pneumonia (Fig. 1). The patient continued to experience headaches after admission, and on the 7th day, the headaches worsened with intermittent throbbing pain. Immediate head CT was performed, but no abnormalities were found (Fig. 2A–C). Meanwhile, a reexamination of the lung CT showed an increase in infiltrative lesions in both lungs compared to the time of admission, progressing from the periphery to the center of both lungs, with over 50% involvement in the lungs. On the 11th day of admission, the patient experienced dizziness and even fell, prompting a second head CT scan (the second since admission), which showed no significant abnormalities except for a vaguely lower density shadow in the right parietal lobe (Fig. 2D–F). On the 13th day of admission, the patient had a sudden seizure lasting about 3 minutes, accompanied by episodic vision loss and hallucinations. The patient underwent a cranial MRI and a lumbar puncture to examine CSF. The MRI showed multiple patchy lesions of high signal intensity in the gray matter and gray-white matter junction of the bilateral frontal, parietal, and occipital lobes, as well as in the brain gyrus on T2 weighted imaging (T2WI) and fluid attenuated inversion recovery (FLAIR) (mainly in the bilateral parietal lobes) (Fig. 3). The patient’s intracranial pressure was not high, and the CSF examination showed no abnormalities in cell count, protein, glucose, chloride, etc. The CSF macro-gene detection showed a positive result for SARS-Cov-2 (with a sequence number of 6), while no bacteria, fungi, or deoxyribonucleic acid viruses were detected. The results of the CSF autoimmune encephalitis antibody test for 6 items (including anti-NMDA receptor antibody, anti-AMPA1 receptor antibody, anti-AMPA2 receptor antibody, anti-GABAB receptor antibody, anti-LGI1 antibody, and anti-CASPR2 antibody) were all negative, and the oligoclonal band test of CSF was negative as well. Based on the patient’s clinical presentation, CSF examination, and brain MRI results, the patient was diagnosed with COVID-19 infection and associated encephalitis. After comprehensive treatment with immunoglobulin infusion (0.4g/kg for 5 days), sustained-release sodium valproate for anti-seizure (0.5 g, twice a day), glycerol fructose for intracranial pressure reduction, Azvudine (FNC) for antiviral therapy (5 mg per dose, once daily, for 14 days), methylprednisolone for anti-inflammation (40 mg/day), and cough relief, the neurological symptoms of the patient improved significantly. On the 25th day of admission, the patient underwent a second brain MRI, which showed that the T2WI and FLAIR high signal lesions in the bilateral frontal, parietal, and occipital lobes had mostly disappeared (Fig. 4). The patient’s CSF routine and biochemical examination were normal upon review, and the nasopharyngeal swab for COVID-19 testing was negative for ORF-1ab and N genes. On the 29th day of admission, the patient’s symptoms were significantly improved, mental state was good, and vital signs were normal, and was discharged on the same day. One month after discharge, the patient returned for follow-up, and the occipital lobe still had a few slow waves on electroencephalogram, but the patient reported no seizure events since discharge.

## 3. Discussion

We reported a case of acute encephalitis with symptoms of dizziness, headache, convulsions, and hallucinations during infection with the SARS-CoV-2 Omicron variant. The patient’s early head CT scan showed no abnormalities, while the head MRI scan showed abnormalities, and SARS-CoV-2 RNA was detected in the CSF. The patient recovered and was discharged after 29 days of timely diagnosis and effective treatment.

Zamani et al^[5]^ summarized 4 potential mechanisms of neuroinflammation during SARS-CoV-2 infection, including; Immune-mediated inflammation and migration of inflammatory cytokines to the central nervous system; Molecular mimicry or immune activity leading to synthesis of CSF or systemic autoantibodies; Direct hematogenous invasion by virus through disruption of the blood-brain barrier; and Direct neuronal invasion through the cribriform plate, olfactory bulb, or other cranial nerves. Direct invasion of the brain by the virus is also one of the above mechanisms but is rare. Some literature reports suggest that a small number of cases exhibit direct invasion of SARS-CoV-2 into brain tissue (detected in CSF or brain tissue biopsy), while most cases with delayed neurological involvement and negative CSF polymerase chain reaction are mainly caused by excessive immune cell reactions.^[5–7]^

The case reported in this article detected SARS-CoV-2 RNA virus in the patient’s CSF, supporting the diagnosis of primary encephalitis, which is inflammation caused by direct viral infection. Its potential mechanism may SARS-CoV-2 has a high affinity for the angiotensin converting enzyme 2 receptor. Angiotensin converting enzyme 2 receptors are widely present in nasal epithelial cells, vascular endothelial cells, brain neurons, astrocytes, and oligodendrocytes. The virus by disrupting the blood-brain barrier guide the bloodline invasion. The patient’s cranial MRI examination showed multiple abnormal signals in the intracranial brain substance. In the context of the global pandemic of COVID-19, complete laboratory analysis of atypical cases is very rare, especially when CSF test results are often negative. Imaging studies plays a crucial role in determining the cause of SARS-CoV-2-related neurological symptoms and is one of the most important diagnostic tools. Kandemirli et al believe that cortical involvement characterized by FLAIR image signal abnormalities, restricted cortical diffusion, and occasional leptomeningeal enhancement on FLAIR sequence enhancement scans may be indicative of SARS-CoV-2 infectious or autoimmune neurological manifestations.^[8]^ In the diagnosis and treatment process of this case, it was shown that MRI is more sensitive than cranial CT in diagnosing early-stage neurological abnormalities in viral infections, especially in the acute phase of viral infection. When other test results are negative, conventional MRI sequences such as T2WI, T2-FLAIR, and DWI can indicate the occurrence of acute encephalitis in COVID-19 patients.

Azvudine (FNC) is a nucleoside analog that inhibits HIV-1 RNA-dependent RNA polymerase (RdRp),^[9]^ In 2022, during the outbreak of the Omicron variant and its branches in China, Azvudine was widely used in clinical treatment of COVID-19 positive patients. The patient we report here had a decrease in white blood cells, red blood cells, and platelet counts on admission, which may have been caused by the “pancytopenia” associated with systemic lupus erythematosus, indicating immune dysfunction. Patients with immune system disorders have a higher chance of developing severe symptoms during infection with the novel coronavirus compared to those without immune disorders.^[10]^ This case continued to progress after admission, as despite the administration of low-dose steroids, Azvudine antiviral treatment and symptomatic treatment, the patient’s pulmonary infiltrates continued to increase, and new neurological symptoms such as dizziness, unsteady gait, falls, seizures, and hallucinations emerged. Therefore, immunoglobulin infusion therapy was added, and the duration of Azvudine use was extended to 14 days. Due to timely treatment, the patient’s clinical symptoms and radiographic findings were significantly improved at discharge. Yu, B et al^[11]^ believe that Azvudine can address COVID-19 infection and may be used to treat future coronavirus infections and other coronavirus-related diseases.

In summary, we report a case of an immunocompromised patient who developed acute encephalitis directly caused by viral invasion of the nervous system following infection with the Omicron variant. This case includes complete imaging data and CSF sequencing results, documenting the evolution of diagnosis and treatment of the disease. This suggests that immunocompromised patients who are unvaccinated should be vigilant for the development of encephalitis following SARS-CoV-2 infection. Patients with SARS-CoV-2 infection-related encephalitis may present with cognitive, psychiatric, consciousness disorders, and seizure. We believe that early detection and appropriate treatment can help patients recover. MRI is helpful in the early diagnosis and efficacy evaluation of SARS-CoV-2-induced encephalitis.

## Author contributions

**Conceptualisation and supervision:** Fanyu Zhao.

**Writing – original draft:** Xiong Zhang.

**Writing – review & editing:** Ruiting Hu.
